# Presenting a Multispectral Image Sensor for Quantification of Total Polyphenols in Low-Temperature Stressed Tomato Seedlings Using Hyperspectral Imaging

**DOI:** 10.3390/s24134260

**Published:** 2024-06-30

**Authors:** Ye Seong Kang, Chan Seok Ryu, Jeong Gyun Kang

**Affiliations:** 1 Department of Smart Agro-Industry, Institute of Agriculture and Life Sciences, Gyeongsang National University, Jinju 52725, Republic of Korea; slow321@gnu.ac.kr; 2 Department of Biosystem Engineering, Institute of Agriculture and Life Sciences, Gyeongsang National University, Jinju 52828, Republic of Korea; 3 DIA Co., Ltd., Jaeju 63169, Republic of Korea

**Keywords:** full width at half maximum, hyperspectral imaging, lasso regression, low-temperature stress, multispectral image sensor, tomato seedling

## Abstract

Hyperspectral imaging was used to predict the total polyphenol content in low-temperature stressed tomato seedlings for the development of a multispectral image sensor. The spectral data with a full width at half maximum (FWHM) of 5 nm were merged to obtain FWHMs of 10 nm, 25 nm, and 50 nm using a commercialized bandpass filter. Using the permutation importance method and regression coefficients, we developed the least absolute shrinkage and selection operator (Lasso) regression models by setting the band number to ≥11, ≤10, and ≤5 for each FWHM. The regression model using 56 bands with an FWHM of 5 nm resulted in an R^2^ of 0.71, an RMSE of 3.99 mg/g, and an RE of 9.04%, whereas the model developed using the spectral data of only 5 bands with a FWHM of 25 nm (at 519.5 nm, 620.1 nm, 660.3 nm, 719.8 nm, and 980.3 nm) provided an R^2^ of 0.62, an RMSE of 4.54 mg/g, and an RE of 10.3%. These results show that a multispectral image sensor can be developed to predict the total polyphenol content of tomato seedlings subjected to low-temperature stress, paving the way for energy saving and low-temperature stress damage prevention in vegetable seedling production.

## 1. Introduction

Vegetable seedling production is an important process that determines successful crop establishment [1] and is a precision industry where cultivation-related technologies such as germination, grafting, growth control, and pest management are employed. Seedling quality is mainly affected by three stress factors: temperature, moisture, and physical stress. Accumulated stress can impair the physiological function of seedlings, reducing their chances of survival and growth [2]. High-temperature stress and moisture in summer and low-temperature stress in winter affect seedling growth [3]. Thus, technologies that can nondestructively detect the quality of seedlings grown under these environmental stresses are required to improve seedling management [4]. In economic terms, photothermal power accounts for the highest proportion of the production cost following seed and labor costs [5]. Therefore, monitoring nondestructive changes in seedling quality can save energy and reduce costs in greenhouse seedling production while identifying environmental conditions suitable for crops [6]. Ultimately, research on seedling quality, growth modeling, and environmental control should be integrated to enhance greenhouse seedling production.

Hyperspectral image sensors are used to observe vegetation growth and physiology because they include the visible as well as the near-infrared light bands. They collect high-dimensional data comprising narrow spectral bands in a wide spectrum, which provides valuable information about vegetation [7]. Although they offer nondestructive quantitative detection of chemical elements, the high dimensionality of the feature space [8] and the large volume of data, including those that are unrelated or redundant [9], can lead to reduced model performance and reproducibility. In addition, a hyperspectral image sensor is difficult to consider as being of a reasonable size and price to be supplied to propagation houses. Therefore, it is an important task to develop a multispectral image sensor of a size and price that is easy to supply while maintaining model performance.

The least absolute shrinkage and selection operator (Lasso) regression is a useful tool to analyze high-dimensional data [10]. Lasso is a shrinkage method that controls model complexity by adding a constraint on the linear model coefficients as a penalty term [11]. Lasso shrinks some regression coefficients to zero for estimation in a linear model and keeps the important features of subset selection [12]. Thus, Lasso has been used to select bands that are sensitive to recognizing the quality of the target vegetation and food [13,14].

Tomato (*Solanum Lycopersicum* L.) is a warm-season crop and is susceptible to cold at all stages of growth [15]. It has been reported that the optimum temperature for leaf growth during the tomato seedling season is 22 °C, the optimum temperature for growth in the entire growing season is between 17 °C and 27 °C, and the upper and lower temperature limits are 10 °C and 35 °C [16]. Inappropriate temperature conditions cause the development of many foliar, stem, and soil-borne plant diseases [17]. Studies have been conducted to observe temperature-induced chemical changes that reflect the quality of tomato seedlings [18]. Tomato seedlings grown under low-temperature stress at 4 °C showed the highest number of phenolic acids and flavonoids, which are some of the polyphenols [19]. These studies verified that the number of chemical components in tomato seedlings is closely related to temperature stress. Polyphenols, the largest group of plant-specific metabolites, were observed as representative substances in this study because they are generally recognized as molecules involved in stress protection in plants [20].

The purpose of this study was to determine the change in total polyphenol content in tomato seedlings upon low-temperature stress and evaluate whether it could be quantitatively predicted using key spectral bands for the development of a multispectral image sensor using hyperspectral imaging.

## 2. Materials and Methods

### 2.1. Experimental Design

**Figure 1:**
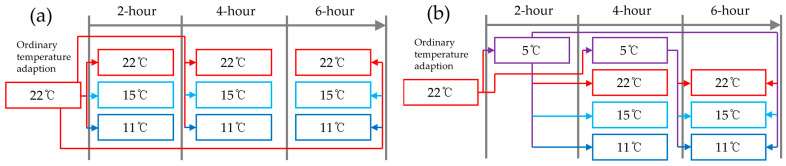
Experiment configuration of (**a**) low-temperature stress treatment and (**b**) low-temperature stress recovery under temperature conditions at (red) 22 °C, (sky blue) 15 °C, (blue) 11 °C, and (purple) 5 °C.

The experiments were conducted at a laboratory at Wonkwang University in January 2017. Tomato seedlings grown for 27 days were exposed to ordinary temperature (22 °C) for 2 h to acclimatize to the same temperature. To observe the effects of low-temperature stress on the polyphenol content, two treatment methods were employed. To observe the effect of low-temperature stress, seedling samples were treated in a chamber maintained at 15 °C and 11 °C compared with the control at 22 °C for up to 6 h, as shown in Figure 1a. For low-temperature stress recovery, tomato seedlings were exposed to low-temperature stress in a chamber maintained at 5 °C for 2 h and 4 h and then re-exposed to 22 °C, 15 °C, and 11 °C, as shown in Figure 1b. Recovery was observed in comparison with the 22 °C sample seedlings (control) every 2 h. Tomato seedlings in all control and treatment groups consisted of 3 samples. The hyperspectral imagery of the seedlings was acquired using the same observation cycle.

### 2.2. Total Polyphenol Content

The total polyphenol contents of tomato seedlings were determined using the Folin–Denis method [21]. A total of 1 mg of the freeze-dried powder was added to 1 mL of 95% ethanol, followed by the addition of 1 mL of Folin–Ciocalteu phenol reagent. Then, they were mixed in a shaking water bath at 27 °C. After 5 min, 1 mL of saturated Na_2_CO_3_ solution was added, mixed, and left at room temperature for 1 h. Consequently, the absorbance was measured at 760 nm using a UV/VIS spectrophotometer (Cary 5000, Varian, Palo Alto, CA, USA). The standard calibration curve was prepared using tannic acid, and the total polyphenol contents of the samples were quantified in terms of conversion.

### 2.3. Hyperspectral Imaging

A hyperspectral image sensor (PS, Specim, Oulu, Finland) comprising 519 spectral bands with a full width at half maximum (FWHM) of 5.5 nm in the wavelength range of 400–1000 nm was used. In addition, the sensor was a lens (OLE 23 C-Mount, Specim, Finland) with a focal length of 23 mm, an aperture of F2.3, and a field of view of 21.1. The images were acquired at a height of 1.5 m above the ground and a shooting angle (rotation angle of the rotator) of 30 degrees with an 18% white reference board (EzyBalance, Lasolite Ltd., Cassola, Italy). To compensate for the noise generated by the heat of the hyperspectral sensor, dark current images were acquired with the closed aperture and preprocessed along with optical correction using the white reference board area as follows:

(1) $$I=\frac{{DN}_{i}}{{DN}_{w}}-{DN}_{d}$$

where I is the corrected hyperspectral image, DN is the digital number indicating the reflectance of all spectra, ${DN}_{i}$ represents the hyperspectral image before correction, ${DN}_{w}$ represents the reflectance of the white reference board area, and ${DN}_{d}$ represents the dark correction image.

**Figure 2:**
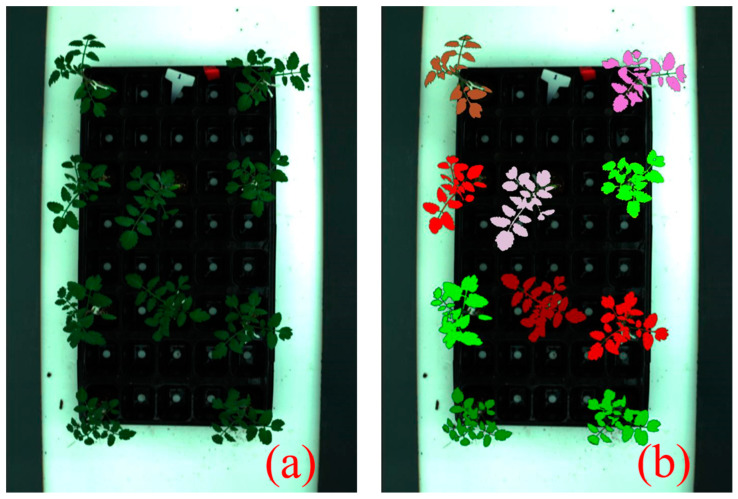
Extraction of the areas of (**a**) individual tomato seedlings after image processing from (**b**) the raw hyperspectral imagery.

GreenNDVI-NDVI was applied to extract the seedling area based on the density slice method using spectral image processing software (ENVI 5.3, Exelis Visual Information Solution Inc., McLean, VA, USA):

(2) $$GreenNDVI-NDVI=\frac{{DN}_{NIR}-{DN}_{Green}}{{DN}_{NIR}+{DN}_{Green}}-\frac{{DN}_{NIR}-{DN}_{Red}}{{DN}_{NIR}+{DN}_{Red}}$$

where ${DN}_{NIR}$ is the reflectance at 820 nm, ${DN}_{Green}$ is the reflectance at 550 nm, and ${DN}_{Red}$ is the reflectance at 620 nm. Figure 2 shows each seedling area extracted using the image processing method.

### 2.4. Statistics

Statistical methods are provided to determine whether low-temperature stress has affected the polyphenol content of tomato seedlings and to quantitatively predict changes in polyphenols.

#### 2.4.1. Two-Sample *t*-Test

A two-sample *t*-test was performed using a statistical package (Python 3.9, Python software foundation, Wilmington, DE, USA) to investigate changes in total polyphenol content with exposure time at low temperature based on the equation:

(3) $$t=\frac{\left({\bar{x}}_{1}-{\bar{x}}_{2}\right)}{\sqrt{s^{2}(\frac{1}{n_{1}}+\frac{1}{n_{2}})}}$$

where $\sqrt{s^{2}(\frac{1}{n_{1}}+\frac{1}{n_{2}})}$ is the standard error. The denominator was calculated after subtracting −1 from the two-sample sizes. The numerator $\left({\bar{x}}_{1}-{\bar{x}}_{2}\right)$ is the process of subtracting and squaring each datum to the average of all data using the variance calculation method. If the deviation between the average value of the data and the value of the comparison target was greater than the standard deviation, it was considered significant.

The two-sample *t*-test was used to determine whether there was a significant difference in polyphenol content between tomato seedlings exposed to low-temperature stress and unstressed seedlings. It was also used to determine whether seedlings recovered from low-temperature stress when re-exposed to ordinary temperature.

#### 2.4.2. Least Absolute Shrinkage and Selection Operator (Lasso) Regression

The Lasso regression model was established by image-based data analysis software (FinePro, Hortizen Co., Ltd., Jinju, Republic of Korea). Lasso regression identifies the variables and their regression coefficients, resulting in a model with minimized prediction error [22]. It shrinks the regression coefficient vector to zero and sets some coefficients equal to zero, resulting in simultaneous estimation and variable selection procedures [23]. Lasso regression uses the L_1 penalty $\sum_{j=1}^{p}\left|\beta_{j}\right|$ with least squares $\sum_{i=1}^{N}(y_{i}-\sum_{j}x_{ij}\beta_{j}{)}^{2}$, as described in Equation (4), defined by [24], and solves the L_1-penalized regression problem by finding β = {βj} to minimize:

(4) $$\sum_{i=1}^{N}(y_{i}-\sum_{j}x_{ij}\beta_{j}{)}^{2}+\lambda \sum_{j=1}^{p}\left|\beta_{j}\right|$$

where λ is the penalty parameter that determines the amount of shrinkage. This affects the selection of important variables. If λ = 0, it is least squares and, if λ is large enough, it shrinks the regression coefficient to zero.

**Figure 3:**
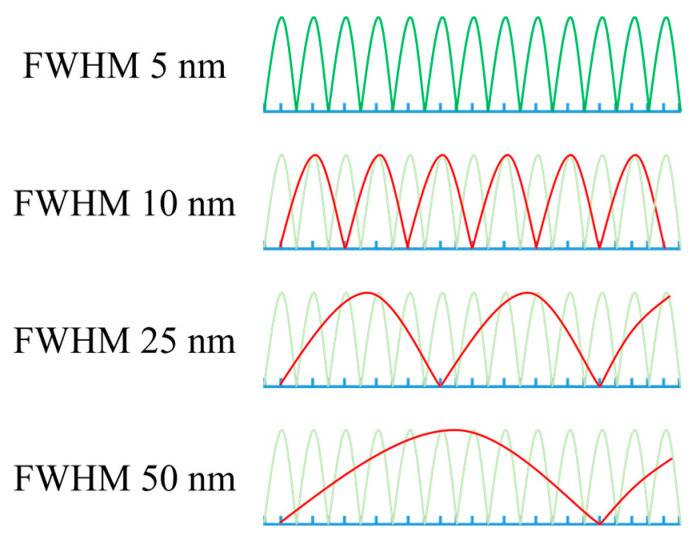
Hyperspectral imagery with an FWHM of 5 nm, which was merged at 10 nm, 20 nm, and 30 nm intervals in the central bands to obtain FWHMs of 10 nm, 25 nm, and 50 nm.

Before performing the Lasso regression analysis, the bandwidth of the hyperspectral imagery with an FWHM of 5 nm was merged at 10 nm, 20 nm, and 30 nm intervals in the central band to obtain FWHMs of 10 nm, 25 nm, and 50 nm using a commercial bandpass filter (lens) mounted on the multispectral image sensor, as shown in Figure 3.

For all FWHMs, regression models were developed and model performance was evaluated. Tenfold validation was used to determine the presence of overfitting. The regression models were evaluated to determine (R^2^), root mean square error (RMSE), and relative error (RE). The RMSE and RE were calculated by Equations (5) and (6), respectively:

(5) $$RMSE=\sqrt{\frac{\sum_{i=1}^{n}(y_{i}-{\hat{y}}_{i}{)}^{2}}{n}}$$

(6) $$RE=\frac{100}{\bar{y}}\sqrt{\frac{\sum_{i=1}^{n}(y_{i}-{\hat{y}}_{i}{)}^{2}}{n}}$$

where $y_{i}$ and ${\hat{y}}_{i}$ are the observed and predicted total polyphenol content, respectively, $\bar{y}$ is the average value, and n is the number of samples.

#### 2.4.3. Band Selection

No more than 10 or 5 key bands were selected at a time to develop a multispectral image sensor, which would be advantageous in terms of processing time and cost [25]. For all FWHM-based regression models, band selection was performed using the permutation importance method. This method offers fast computation because it does not retrain the model and can be applied to any model and can measure the importance of consistent variables. Finally, after selecting key bands by comparing them with Lasso regression coefficients and developing a regression model composed of only those bands, the possibility of developing a multispectral image sensor based on 10 or 5 bands or less was evaluated in comparison with the regression model based on the full spectral wavelength range.

## 3. Results and Discussion

### 3.1. Changes in Total Polyphenol Content and Spectral Features

Table 1 presents the two-sample *t*-test results with the mean ± standard deviation of total polyphenol for the control (22 °C) and treatment (15 °C and 11 °C) groups based on exposure time (2 h, 4 h, and 6 h). For all exposure time conditions, it showed the lowest total polyphenol content with a significant difference only at 22 °C. There was no significant difference between 15 °C and 11 °C; thus, it was considered that they had the same low-temperature stress effect. Compared with the control group, the total polyphenol content in the low-temperature stress treatment group was about 15% = −19%, 20%, and 15% for exposure times of 2, 4, and 6 h, respectively. The total polyphenol content increased less with the increased exposure time because of the developed resistance to low-temperature stress, which did not deviate from the critical low-temperature stress [26].

Table 2 presents the two-sample *t*-test results with the mean ± standard deviation of total polyphenol in which seedlings were re-exposed to 22 °C, 15 °C, and 11 °C after exposure to 5 °C for 2 or 4 h. In the case of the seedlings exposed to 5 °C for 2 h and re-exposed to different temperatures for 2 h, there was no significant difference in the total polyphenol content from that of the control group upon re-exposure to 22 °C, whereas the total polyphenol content was remarkably higher than that of the control group upon re-exposure to 15 °C and 11 °C. This means that it was recovered only at 22 °C. For the seedlings exposed at 5 °C for 2 h and re-exposed to different temperatures for 4 h, the total polyphenol contents of the re-exposed seedlings were significantly different from that of the control group, so they were considered not recovered in these conditions. Upon re-exposure to 11 °C, the polyphenol content was higher than that obtained upon re-exposure to other temperatures, indicating that the effect of low-temperature stress was greater. Overall, compared with the control group, the total polyphenol content increased at a low temperature and recovery became more difficult as the exposure time increased.

**Figure 4:**
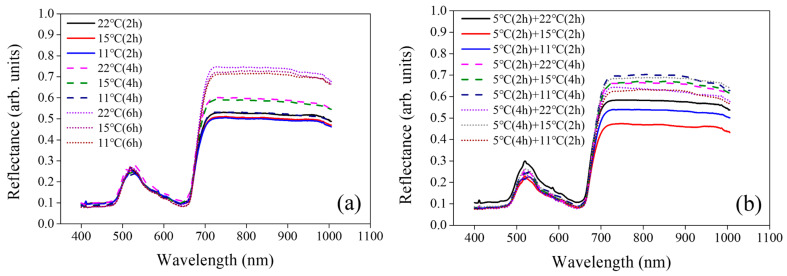
Averaged reflectance curves for each treatment group upon (**a**) low-temperature stress and (**b**) stress recovery versus wavelength.

Figure 4a shows the reflectance curves of tomato seedlings averaged for each temperature and exposure time condition. The reflectance curve showed a clear upward trend at the red edge (from about 650 nm to 720 nm) and NIR range (from about 720 nm to 1000 nm) for each temperature and exposure time, indicating that the hyperspectral image sensor can detect the response of seedlings to low-temperature stress [27].

Figure 4b shows the reflectance curves of tomato seedlings averaged for each temperature and exposure time condition upon stress recovery. As shown in the two-sample *t*-test results, the reflectance decreased in the visible-light wavelength region (~<650 nm), while it increased in the wavelength region after the red edge (~>650 nm), depending on the degree of recovery after exposure to 5 °C.

### 3.2. Quantitative Prediction of Total Polyphenols Using Key Spectral Bands

#### 3.2.1. Selection of Key Spectral Bands

**Figure 5:**
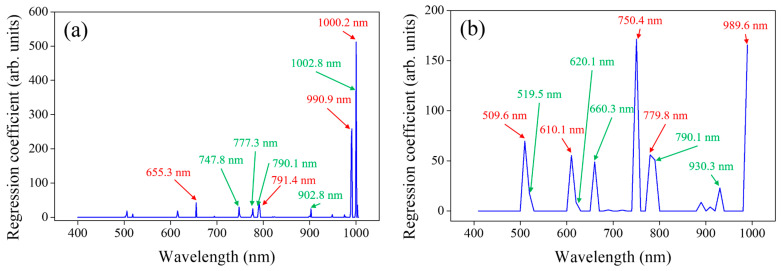

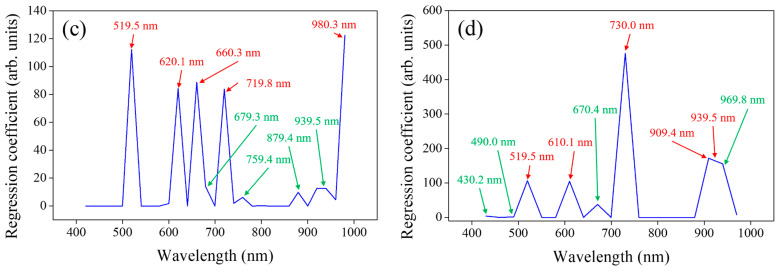
Central regression coefficient bands with band numbers of ≤10 (green and red) and ≤5 (red) and an FWHM of (**a**) 5 nm, (**b**) 10 nm, (**c**) 25 nm, and (**d**) 50 nm.

In general, to develop a multispectral image sensor, 5 or less than 10 central bands with an FWHM of 10 nm or more should be selected. Figure 5 shows the regression coefficients depending on wavelength based on the Lasso regression models for each FWHM.

Figure 5a shows the key spectral bands sensitive to the prediction of total polyphenol content in tomato seedlings at an FWHM of 5 nm. The green and red colors indicate the cases where less than 10 bands were selected and the red color indicates the cases where less than 5 bands were selected. When less than 10 bands were selected, adjacent bands (such as 790.1 nm and 791.4 nm, and 1000.2 nm and 1002.8 nm) were selected. In addition, only the central bands above the red edge were selected, which was considered disadvantageous for use other than the low-temperature stress of tomato seedlings [28]. When reducing the band number to 5 or less, adjacent bands were removed and only the central bands corresponding to the peaks in each regression coefficient were selected. In the NIR band over 900 nm, the regression coefficient was overwhelmingly high.

Figure 5b shows the key spectral bands sensitive to the prediction of total polyphenol content in tomato seedlings at an FWHM of 10 nm. The selected spectral bands included two green (509.6 nm and 519.5 nm), two red (601.1 nm and 620.1 nm), one red edge (660.3 nm), three NIR bands below 900 nm (750.4 nm, 779.8 nm, and 790.1 nm), and two NIR bands over 900 nm (930.3 nm and 989.6 nm). In contrast to the case of a 5 nm FWHM, the central bands at various wavelengths were selected since it is more advantageous in terms of sensor application variety. When less than 5 bands were selected, the red edge was not selected and three NIR bands were selected the most, with a high regression coefficient.

Figure 5c shows the key spectral bands sensitive to the prediction of total polyphenol content in tomato seedlings at an FWHM of 25 nm. One green (519.5 nm), one red (620.1 nm), three red edge (660.3 nm, 679.3 nm, and 719.8 nm), two NIR bands below 900 nm (759.4 nm and 879.4 nm), and two NIR bands over 900 nm (939.5 nm and 980.3 nm) were selected. Unlike the cases of FWHMs of 5 nm and 10 nm, the number of red edge bands was the highest. Even when only five bands were selected, one green, one red, one NIR band, and two red edge bands were selected in a balanced way with similar regression coefficients, unlike in other FWHM conditions. In the case of tea leaves, it has been reported that the red edge band of 660 nm and NIR bands over 900 nm played an important role in predicting polyphenol content, just like the bands selected at FWHM of 25 nm [29].

Figure 5d shows the key spectral bands sensitive to the prediction of total polyphenol content in tomato seedlings at an FWHM of 50 nm. Two blue (430.2 nm and 490.0 nm), one green (519.5 nm), one red (610.1 nm), two red edge (670.4 nm and 730.0 nm), and NIR bands over 900 nm (909.4 nm, 939.5 nm, and 969.8 nm) were selected. Unlike bands in other FWHM cases, two blue bands were selected but removed when five bands were selected. It has been reported that total polyphenols and pigments such as chlorophyll and carotenoids are affected by high inverse correlation due to low temperature stress.

When exposed to low temperature stress, the total polyphenol content of seedlings increases, while the chlorophyll content significantly decreases and carotenoids remain relatively unchanged [30]. Thus, it can be determined that the green band, which is a wavelength that reflects sensitively to changes in chlorophyll, and the red band, which is an absorbing wavelength, have a higher impact than the blue bands. Red edge has been frequently used to evaluate the effects of stress on plants [31], and NIR between 900 and 1000 nm has played a crucial role in quantifying substances at the molecular level [32]. However, the NIR band over 900 nm, which had a relatively high regression coefficient in other FWHMs, showed a relatively low regression coefficient. It is necessary to determine how the bands selected with each FWHM affect the performance of the regression model.

#### 3.2.2. Prediction Performance of Lasso Regression Models

Table 3 presents the performance of Lasso regression models depending on the number (≥11, ≤10, and ≤5) of spectral bands selected to predict the total polyphenol content of low-temperature-stressed tomato seedlings using hyperspectral imaging. When the Lasso regression models were established using the full bands at each FWHM, 56, 15, and 14 bands were selected for FWHMs of 5 nm, 10 nm, and 25 nm, respectively. Since only 9 bands were selected for an FWHM of 50 nm, the performance was not indicated for the band number ≥ 11. The prediction model for an FWHM of 5 nm resulted in R^2^ = 0.71, RMSE = 3.99 mg/g, and RE = 9.04%, and the validation model gave R^2^ = 0.50, RMSE = 5.25 mg/g, and RE = 11.9%. The prediction models for FWHMs of 10 nm and 25 nm led to R^2^ ≥ 0.64, RMSE ≤ 4.46 mg/g, and RE ≤ 10.1%, and the validation models resulted in R^2^ ≥ 0.45, RMSE ≤ 5.51 mg/g, and RE ≤ 12.5%. Compared with the case of a 5 nm FWHM, the number of bands decreased from 56 to less than 15, but the prediction performance slightly decreased. As shown in Figure 2, this is because the central bands include the spectral attributes of adjacent bands as the bandwidth becomes broader [33].

Conversely, when the number of bands at an FWHM of 5 nm was less than 10 and 5, the prediction performance decreased sharply to R^2^ ≥ 0.41, RMSE ≤ 5.69 mg/g, and RE ≤ 12.9% because it did not include the spectral attributes of adjacent bands and involved information only in a narrow wavelength range. When the model was developed by selecting less than 10 bands with an FWHM of 10 nm, the prediction model provided R^2^ = 0.66, RMSE = 4.35 mg/g, and RE = 9.83% and the validation model gave R^2^ = 0.49, RMSE = 5.31 mg/g, and RE = 12.0%. It showed similar performance for an FWHM of 25 nm but, for an FWHM of 50 nm, unlike other FWHMs, it showed slightly reduced performance along with the relatively low NIR regression coefficient trend because of the congested spectral information in the broader wavelength region, as shown in Figure 5.

When regression models were developed using the most advantageous five bands for developing a multispectral image sensor, for an FWHM of 25 nm, the prediction performance of the regression model was the highest, with R^2^ = 0.62, RMSE = 4.54 mg/g, and RE = 10.3%. The performance of the validation model was similar to that of the validation model developed using 56 bands with an FWHM of 5 nm. These results showed the possibility of predicting the total polyphenol content of tomato seedlings using only 5 bands. In addition, the regression models using an FWHM of 25 nm maintained their performance regardless of the band number with a linearity of >62% and an error of <4.54 mg/g. It may be due to the influence of bands selected in a balanced way with similar regression coefficients, as shown in Figure 5c. The visible-light peak at 519.5 nm, shown in Figure 4, corresponds to leaf reflectance and color-related leaf properties affected by chlorophyll a, chlorophyll b, and β-carotene [34]. Since chlorophyll and polyphenols can be used as nitrogen indicators, the peak can be correlated with polyphenols [35,36]. The 660.3 nm wavelength is close to the starting point of the red edge and 719.8 nm is the endpoint of the red edge. The red edge indicates a sharp change in vegetative chlorophyll and nitrogen [37,38]. Thus, it also means that the selected band can be correlated with the changes in the total polyphenol content of tomato seedlings. Finally, the 980.3 nm band with the highest regression coefficient in the NIR, which dominates the moisture content of leaves, was used for predicting the chemical information of vegetation [39]. In addition, the water absorption band is advantageous for distinguishing drought-stressed vegetation through water content prediction [40]. To reduce the sensor cost and size through multipurpose use, it is also important to verify that it can contribute to saving energy and time and amount of control by predicting the moisture content of heating and drought-stressed tomato seedlings using the key bands demonstrated in this study.

**Figure 6:**
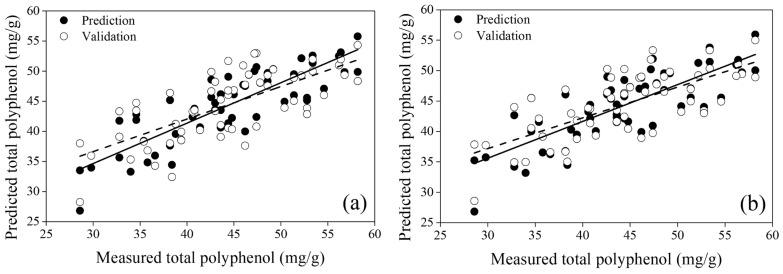
Relationship between the predicted and measured total polyphenol contents based on Lasso regression using (**a**) 56 bands with an FWHM of 5 nm and (**b**) 5 bands with an FWHM of 25 nm.

Figure 6 shows the linear relationship between the regression model using 56 bands with an FWHM of 5 nm and that using only 5 bands with an FWHM of 25 nm. Both plots show adequate linearity, with errors as low as 10%. The results indicate that the polyphenol quantification model of tomato seedlings can be used to monitor temperature and observe the total polyphenol change in real time using a cost-effective multispectral image sensor, paving the way for energy saving and stress-induced damage prevention.

**Figure 7:**
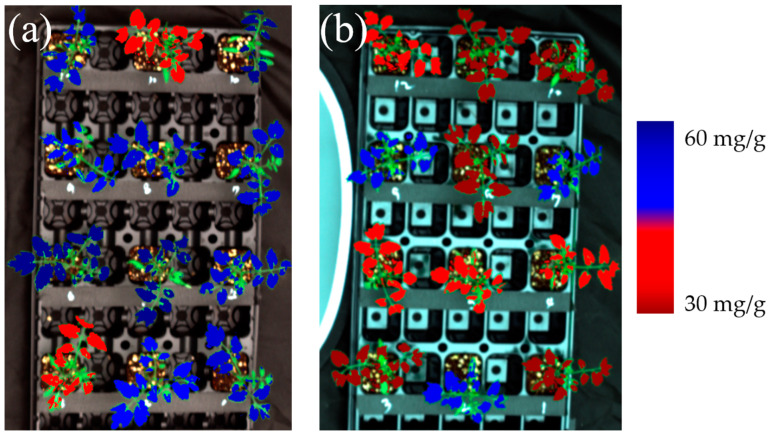
Distribution of total polyphenol content predicted by Lasso regression model using 5 bands with an FWHM of 25 nm of (**a**) tomato seedlings exposed at 11 °C and (**b**) tomato seedlings exposed at 22 °C for 6 h.

Figure 7 shows the distribution of total polyphenol content of tomato seedlings exposed to 11 °C (Figure 1a) and 22 °C (Figure 1b) for 6 h, respectively. The total polyphenol content distribution results were predicted by a Lasso regression model using five bands with an FWHM of 25 nm. As shown in Table 1, it was determined that tomato seedlings exposed to 11 °C had generally higher total polyphenol content than those exposed to 22 °C.

## 4. Conclusions

Hyperspectral imaging was used to demonstrate the predictability of the total polyphenol content in low-temperature-stressed tomato seedlings. To nondestructively detect low-temperature stress, the Lasso regression model was established with the selection of key bands using hyperspectral image data to quantify the total polyphenol content of tomato seedlings. First, the spectral data with an FWHM of 5 nm were merged to obtain FWHMs of 10 nm, 25 nm, and 50 nm using a commercialized bandpass filter to test the potential for the development of a cost-effective multispectral image sensor. The Lasso regression model developed using only 5 bands with an FWHM of 25 nm suggests the feasibility of the development of a cheaper multispectral image sensor. Because multispectral imaging technology can be used over time change in the fall and winter to establish the lowest temperature conditions possible for tomato seedlings to resist low temperatures, it can provide energy saving by reducing heating and prevent stress-induced damage in greenhouse seedling production. However, the RE in the validation model was less than 12%, but R^2^ was 0.50, so it is necessary to increase the linearity of the validation model by adding samples through further experiments. Nevertheless, this study is considered significant in that it selected key spectral bands that respond sensitively to polyphenol changes and presented a specific analysis method to select the key spectral bands for the development of a multispectral image sensor based on hyperspectral image data.

## Figures and Tables

**Table 1:** Variation in total polyphenol content in low-temperature-stressed tomato seedlings with exposure time.

	Total Polyphenol (mg/g)		
	2 h	4 h	6 h
22 °C	37.38 ± 2.50 ^a 1^	30.39 ± 2.16 ^a^	36.78 ± 1.51 ^a^
15 °C	45.98 ± 4.69 ^b^	37.87 ± 1.93 ^b^	43.58 ± 1.93 ^b^
11 °C	43.78 ± 2.75 ^b^	37.78 ± 3.02 ^b^	48.18 ± 5.17 ^b^

^1^ Two-sample *t*-test at the significance level (*p*-value < 0.05) with mean ± standard deviation.

**Table 2:** Variation of total polyphenol content in tomato seedlings with recovery temperature and time following low-temperature stress treatment.

Stress Temperature	Stress Time	Recovery Temperature	Recovery (Treatment) Time	Total Polyphenol (mg/g)
-	0	22 °C (Control)	4	**30.39 ± 2.16** ***^a^*** ^1^
5 °C	2	22 °C	2	**31.79 ± 2.83** ***^a^***
		15 °C		**53.58 ± 1.20** ***^b^***
		11 °C		**53.65 ± 6.20** ***^b^***
-	0	22 °C (Control)	6	**36.78 ± 1.51** ***^a^***
5 °C	2	22 °C	4	**44.19 ± 3.30** ***^b^***
		15 °C		**44.79 ± 2.42** ***^b^***
		11 °C		**45.38 ± 2.16** ***^b^***
-	0	22 °C (Control)	6	**36.78 ± 1.51** ***^a^***
5 °C	4	22 °C	2	**46.98 ± 1.39** ***^b^***
		15 °C		**50.58 ± 2.11** ***^b^***
		11 °C		**55.98 ± 2.42** ***^c^***

^1^ Two-sample *t*-test at the significance level (*p*-value < 0.05) with mean ± standard deviation.

**Table 3:** Lasso regression models for each FWHM based on the band number (≥11, ≤10, and ≤5).

		FWHM—5 nm	FWHM—10 nm	FWHM—25 nm	FWHM—50 nm
Number of bands (≥11)		56	15	14	
Prediction	R^2^	0.71	0.66	0.64	
	RMSE (mg/g)	3.99	4.33	4.46	
	RE (%)	9.04	9.79	10.1	
Validation	R^2^	0.50	0.47	0.45	
	RMSE (mg/g)	5.25	5.45	5.51	
	RE (%)	11.9	12.3	12.5	
Number of bands (≤10)		9	10	9	9
Prediction	R^2^	0.58	0.66	0.64	0.59
	RMSE (mg/g)	4.86	4.35	4.47	4.76
	RE (%)	11.0	9.83	10.1	10.8
Validation	R^2^	0.46	0.49	0.49	0.39
	RMSE (mg/g)	5.48	5.31	5.33	5.81
	RE (%)	12.4	12.0	12.1	13.1
Number of bands (≤5)		4	5	5	5
Prediction	R^2^	0.41	0.57	0.62	0.57
	RMSE (mg/g)	5.69	4.91	4.54	4.87
	RE (%)	12.9	11.1	10.3	11.0
Validation	R^2^	0.30	0.43	0.50	0.45
	RMSE (mg/g)	6.22	5.62	5.27	5.51
	RE (%)	14.1	12.7	11.9	12.5

## Data Availability

Data are contained within the article.

## References

[1] Bantis F., Koukounaras A., Siomos A.S., Dangitsis C. (2020). Impact of scion and rootstock seedling quality selection on the vigor of watermelon–interspecific squash grafted seedlings. Agriculture.

[2] Haase D.L., Riley L.E., Dumroese R.K., Landis T.D. (2007). Morphological and physiological evaluations of seedling quality. National Proceedings Forest and Conservation Nursery Associations Proceedings RMRS-P-50 2006.

[3] DiPaola J.M., Beard J.B. (1992). Physiological effects of temperature stress. Agron. Monogr..

[4] Zhang M., Zhang W., Chen X., Wang F., Wang H., Zhang J., Liu L. (2021). Modeling and simulation of temperature control system in plant factory using energy balance. Int. J. Agric. Biol. Eng..

[5] Lewis M., Kubota C., Tronstad R., Son Y.J. (2014). Scenario-based cost analysis for vegetable grafting nurseries of different technologies and sizes. HortScience.

[6] Kang J.G., Ryu C.S., Kim S.H., Kang Y.S., Sarkar T.K., Kang D.H., Kim D.E., Ku Y.G. (2016). Estimating moisture content of cucumber seedling using hyperspectral imagery. J. Biosyst. Eng..

[7] Kang Y.S., Ryu C., Suguri M., Park S.B., Kishino S., Onoyama H. (2022). Estimating the catechin concentrations of new shoots in green tea fields using ground-based hyperspectral imagery. Food Chem..

[8] Sabale S.P., Jadhav C.R. Hyperspectral image classification methods in remote sensing-a review. Proceedings of the International Conference on Computing Communication Control and Automation 2015.

[9] Salimi A., Ziaii M., Amiri A., Hosseinjani Zadeh M.H., Karimpouli S., Moradkhani M. (2018). Using a Feature Subset Selection method and Support Vector Machine to address curse of dimensionality and redundancy in Hyperion hyperspectral data classification. Egypt. J. Remote Sens. Space Sci..

[10] Song Q. (2018). An overview of reciprocal L 1-regularization for high dimensional regression data. Wiley Interdiscip. Rev. Comput. Stat..

[11] Cui C., Wang D. (2016). High dimensional data regression using Lasso model and neural networks with random weights. Inf. Sci..

[12] Araveeporn A. (2021). The higher-order of adaptive lasso and elastic net methods for classification on high dimensional data. Mathematics.

[13] Liu W., Zeng S., Wu G., Li H., Chen F. (2021). Rice seed purity identification technology using hyperspectral image with lasso logistic regression model. Sensors.

[14] Wu J., Ouyang Q., Park B., Kang R., Wang Z., Wang L., Chen Q. (2022). Physicochemical indicators coupled with multivariate analysis for comprehensive evaluation of matcha sensory quality. Food Chem..

[15] Suchoff D.H., Perkins-Veazie P., Sederoff H.W., Schultheis J.R., Kleinhenz M.D., Louws F.J., Gunter C.C. (2018). Grafting the indeterminate tomato cultivar moneymaker onto multifort rootstock improves cold tolerance. HortScience.

[16] Sato S., Kamiyama M., Iwata T., Makita N., Furukawa H., Ikeda H. (2006). Moderate increase of mean daily temperature adversely affects fruit set of Lycopersicon esculentum by disrupting specific physiological processes in male reproductive development. Ann. Bot..

[17] Kumar S.P., Srinivasulu A., Babu K.R. (2018). Symptomology of major fungal diseases on tomato and its management. J. Pharmacogn. Phytochem..

[18] Rivero R.M., Ruiz J.M., Garcıa P.C., López-Lefebre L.R., Sánchez E., Romero L. (2001). Resistance to cold and heat stress: Accumulation of phenolic compounds in tomato and watermelon plants. Plant Sci. Int. J. Exp. Plant Biol..

[19] Alhaithloul H.A.S., Galal F.H., Seufi A.M. (2021). Effect of extreme temperature changes on phenolic, flavonoid contents and antioxidant activity of tomato seedlings (*Solanum Lycopersicum* L.). PeerJ.

[20] Šamec D., Karalija E., Šola I., Vujčić Bok V., Salopek-Sondi B. (2021). The role of polyphenols in abiotic stress response: The influence of molecular structure. Plants.

[21] Folin O., Denis W. (1915). A colorimetric method for determination of phenols (phenol derivatives) inurine. J. Biol. Chem..

[22] Ranstam J., Cook J.A. (2018). Lasso regression. Br. J. Surg..

[23] Hans C. (2009). Bayesian lasso regression. Biometrika.

[24] Tibshirani R. (1996). Regression shrinkage and selection via the lasso. J. R. Stat. Soc. Ser. B.

[25] Pu H., Kamruzzaman M., Sun D.W. (2015). Selection of feature wavelengths for developing multispectral imaging systems for quality, safety and authenticity of muscle foods-a review. Trends Food Sci. Technol..

[26] Schöner S., Heinrich Krause G. (1990). Protective systems against active oxygen species in spinach: Response to cold acclimation in excess light. Planta.

[27] Yang W., Yang C., Hao Z., Xie C., Li M. (2019). Diagnosis of plant cold damage based on hyperspectral imaging and convolutional neural network. IEEE Access.

[28] Kim M.S., Lefcourt A.M., Chao K., Chen Y.R., Kim I., Chan D.E. (2002). Multispectral detection of fecal contamination on apples based on hyperspectral imagery: Part I. Application of visible and near–infrared reflectance imaging. Trans. ASAE.

[29] Xiong C., Liu C., Pan W., Ma F., Xiong C., Qi L., Feng C., Xuzhaong L., Jianbo Y., Zheng L. (2015). Non-destructive determination of total polyphenols content and classification of storage periods of Iron Buddha tea using multispectral imaging system. Food Chem..

[30] Abd Elbar O.H., Elkelish A., Niedbała G., Farag R., Wojciechowski T., Mukherjee S., Abou-Hadid A.F., El-Hennawy H.M., El-Yazied A.A., El-Gawad H.G. (2021). Protective effect of γ-aminobutyric acid against chilling stress during reproductive stage in tomato plants through modulation of sugar metabolism, chloroplast integrity, and antioxidative defense systems. Front. Plant Sci..

[31] Zarco-Tejada P.J., Pushnik J.C., Dobrowski S., Ustin S.L. (2003). Steady-state chlorophyll a fluorescence detection from canopy derivative reflectance and double-peak red-edge effects. Remote Sens. Environ..

[32] Fatchurrahman D., Nosrati M., Amodio M.L., Chaudhry M.M.A., de Chiara M.L.V., Mastrandrea L., Colelli G. (2021). Comparison performance of visible-nir and near-infrared hyperspectral imaging for prediction of nutritional quality of goji berry (*Lycium barbarum* L.). Foods.

[33] Kang Y.S., Jang S.H., Park J.W., Song H.Y., Ryu C.S., Jun S.R., Kim S.H. (2020). Yield prediction and validation of onion (*Allium cepa* L.) using key variables in narrowband hyperspectral imagery and effective accumulated temperature. Comput. Electron. Agric..

[34] Mishra P., Asaari M.S.M., Herrero-Langreo A., Lohumi S., Diezma B., Scheunders P. (2017). Close range hyperspectral imaging of plants: A review. Biosyst. Eng..

[35] Demotes-Mainard S., Boumaza R., Meyer S., Cerovic Z.G. (2008). Indicators of nitrogen status for ornamental woody plants based on optical measurements of leaf epidermal polyphenol and chlorophyll contents. Sci. Hortic..

[36] Gabriel J.L., Quemada M., Alonso-Ayuso M., Lizaso J.I., Martín-Lammerding D. (2019). Predicting N status in maize with clip sensors: Choosing sensor, leaf sampling point, and timing. Sensors.

[37] Horler D.N.H., Dockray M., Barber J. (1983). The red edge of plant leaf reflectance. Int. J. Remote Sens..

[38] Ramoelo A., Skidmore A.K., Cho M.A., Schlerf M., Mathieu R., Heitkönig I.M.A. (2012). Regional estimation of savanna grass nitrogen using the red-edge band of the spaceborne RapidEye sensor. Int. J. Appl. Earth Obs. Geoinf..

[39] Chaudhry M.M.A., Amodio M.L., Amigo J.M., de Chiara M.L.V., Babellahi F., Colelli G. (2020). Feasibility study for the surface prediction and mapping of phytonutrients in minimally processed rocket leaves (*Diplotaxis tenuifolia*) during storage by hyperspectral imaging. Comput. Electron. Agric..

[40] Susič N., Žibrat U., Širca S., Strajnar P., Razinger J., Knapič M., Vončina A., Urek G., Gerič Stare B.G. (2018). Discrimination between abiotic and biotic drought stress in tomatoes using hyperspectral imaging. Sens. Actuators. Part B Chem..

